# PErspective and current status of Radiotherapy Service in IRan (PERSIR)-1 study: assessment of current external beam radiotherapy facilities, staff and techniques compared to the international guidelines

**DOI:** 10.1186/s12885-024-12078-z

**Published:** 2024-03-08

**Authors:** Arefeh Saeedian, Fatemeh-sadat Tabatabaei, Amirali Azimi, Mohammad Babaei, Marzieh Lashkari, Ebrahim Esmati, Zeinab Abiar, Leila Moadabshoar, Saleh Sandoughdaran, Mitchell Kamrava, Arya Amini, Reza Ghalehtaki

**Affiliations:** 1 Radiation Oncology Research Center, Cancer Research Institute, Tehran University of Medical Sciences, Tehran, Iran; 2 Department of Radiation Oncology, Cancer Institute, IKHC, Tehran University of Medical Sciences, Tehran, Iran; 3 St Luke’s Cancer Centre, Royal Surrey County Hospital, Guildford, UK; 4 Department of Radiation Oncology, Cedars Sinai Medical Center, Los Angeles, CA 90048 USA; 5 Department of Radiation Oncology, City of Hope National Medical Center, Duarte, CA 91010 USA; 6 Radiation Oncology Research Center, Radio-Oncology Ward, Cancer Institute, Keshavarz Blvd, Qarib Street, Tehran, Iran

**Keywords:** Radiation oncology, Iran, Cancer care facilities, Developing countries, Services accessibility, Global health

## Abstract

**Background and purpose:**

Radiotherapy (RT) is an essential treatment modality against cancer and becoming even more in demand due to the anticipated increase in cancer incidence. Due to the rapid development of RT technologies amid financial challenges, we aimed to assess the available RT facilities and the issues with achieving health equity based on current equipment compared to the previous reports from Iran.

**Materials and methods:**

A survey arranged by the Iran Cancer Institute's Radiation Oncology Research Center (RORC) was sent to all of the country's radiotherapy centers in 2022. Four components were retrieved: the reimbursement type, equipment, human resources, and patient load. To calculate the radiotherapy utilization rate (RUR), the Lancet Commission was used. The findings were compared with the previous national data.

**Results:**

Seventy-six active radiotherapy centers with 123 Linear accelerators (LINACs) were identified. The centers have been directed in three ways. 10 (20 LINACs), 36 (50 LINACs), and 30 centers (53 LINACs) were charity-, private-, and public-based, respectively. Four provinces had no centers. There was no active intraoperative radiotherapy machine despite its availability in 4 centers. One orthovoltage X-ray machine was active and 14 brachytherapy devices were treating patients. There were 344, 252, and 419 active radiation oncologists, medical physicists, and radiation therapy technologists, respectively. The ratio of LINAC and radiation oncologists to one million populations was 1.68 and 4.10, respectively. Since 2017, 35±5 radiation oncology residents have been trained each year.

**Conclusion:**

There has been a notable growth in RT facilities since the previous reports and Iran's situation is currently acceptable among LMICs. However, there is an urgent need to improve the distribution of the RT infrastructure and provide more facilities that can deliver advanced techniques.

## Introduction

The number of new cancer cases was 19.3 million people worldwide in 2020, which is expected to reach 24.6 million in 2030, suggesting an increase of 27.5% [1]. Developing countries account for around 60% of all new cancer cases and 70% of all cancer-related deaths globally [2]. In Iran, cancer is the third most common cause of disease-related mortality [3]. In 2020, the crude cancer incidence rate was 156.2 cases per 100,000 people in Iran. The numbers are projected to rise from around 131,000 to 184,000 between 2020 and 2030. This growth rate of 39% is higher than that expected in Asia (29%) and the world (25%) [1]. Besides the expected population aging, increased exposure to air pollution and other environmental carcinogens, a sedentary lifestyle, Western pattern diets, and smoking have led to a higher expected cancer rate [4].

Appropriate cancer care services must be readily available to accommodate the expected increase in cancer cases to provide adequate and acceptable patient care. Radiation therapy is an essential treatment that will be used, at some point, in over half of all cancer patients. Nevertheless, cancer treatment is a complicated and costly process, given the psychological, social, and economic pressure on patients and their families [5]. Radiation therapy (RT), which is recognized as a complex treatment, is also an effective and standard modality, regardless of the socioeconomic and cultural contexts of a country [6, 7].

Iran last time reported data on its RT resources in 2015 [8]. In order to ensure that patients have the appropriate access to therapeutic irradiation, it is crucial to identify and understand the current resources and gaps in radiotherapy. Therefore, the present study, which was conducted by the Radiation Oncology Research Center (RORC) of the Iran Cancer Institute, the leading organization for cancer treatment, education, and research in Iran and also the oldest in the Middle East since 1949 [9], aimed to overview Iran's RT current status and its challenges of achieving health equity.

### Materials and methods

This observational cross-sectional study was conducted by the RORC of the Iran Cancer Institute, affiliated with the Tehran University of Medical Sciences, Tehran, Iran. The Institutional Ethics committee has approved this study (IR.TUMS.IKHC.REC.1402.076).

#### Data collection and resources

A survey questionnaire was developed to collect data on radiotherapy centers in all 31 provinces of Iran. The Radiation Oncology Research Center (RORC) vice-director served as the primary correspondent to collect all data and confirm their validity. Data collection began on October 1, 2022, and the goal response rate of 80% of RT centers in all 31 provinces was achieved on November 11, 2022. The survey questionnaire was distributed to each radiotherapy center through a visit from the RT center. In some provinces where it was not feasible to visit the centers in person, the questionnaire was distributed by phone or email. If the response was not achieved, the research team contacted a designated contact person in the province who was a full-time radiation oncologist at that center. The contact person was responsible for contacting all the centers in their province to collect the questionnaire. In order to maintain data quality and accuracy, we double-checked all the data and resolved any discrepancies by reaching out to the responsible person at each center. Overall, the data collection process was designed to ensure that all radiotherapy centers in all 31 provinces were represented in the survey and that the data collected were reliable and accurate.

The questionnaire consisted of four parts: (1) radiotherapy equipment including linear accelerators and brachytherapy machines, orthovoltage X-ray and intra-operative radiotherapy devices, (2) radiotherapy staff, (3) the number of patients treated, and (4) the centers' payment management type.

#### Radiotherapy equipment

The number of active radiotherapy machines was collected according to their types for each center. Since the cobalt-60 system has not been used since 2020 in Iran, it was not included in this study's equipment survey. Data on intraoperative radiation therapy (IORT), orthovoltage, and brachytherapy (BT) units were also included in the study.

#### Radiotherapy staff

The number of radiation oncologists and radiation oncology residents, medical physicists, and radiation therapy technologists was obtained for each center. However, the present study did not investigate the number of nurses and general practitioners. We also received information from the Iranian Society of Radiation Oncology and Society of Medical Physics and Society of Radiation Therapy Technologists of their active members. The staff who were working in more than one center were checked to remove any redundancy. The center with the highest time spent was considered the main workplace for a full-time equivalent worker. The number of radiation oncology residents were checked according to the capacity of programs announced during national resident matching examination.

#### Patient load

In each center, the number of treatment courses and treated patients were retrieved from the center's director. Most high-volume centers presented the actual number during 2022. However, data regarding the number of treatment courses was available monthly for the remaining centers. Thus, the number of treatment courses was estimated based on the working hours of each center per week, month, and year to reduce bias. The total number of treatment courses in the country in 2022 was then calculated. Patients' details were unavailable according to gender, treatment intentions (palliative, adjuvant, or definitive), fractionation (hypo- or hyper- or altered fractionation schedules), or type of cancer in any of the centers.

#### Radiotherapy expense and payments

Payment management types included government (public), private, and charity-based. The government constructs public centers, and most are affiliated with medical universities in the same or nearby city. Basic social health insurance organizations cover 90-100% of all expenses in public centers [10]. The private sector directs private centers, and tariffs are three times the public centers; it is up to each private center to accept public insurance companies. However, most private centers accept insurance companies of the armed forces, Banks, and private insurance companies. Finally, charity-based centers were equipped with donations to charity institutes, and their tariffs are usually 0.75 times the private centers. Basic social health insurance organizations cover about 30% of expenses in private-based centers. The remaining costs in public- and private-based centers are paid by private insurance based on their contract rules. Regarding charity-based centers the basic social health insurance companies cover the expenses equal to a public center, so the remaining costs should be afforded out-of-pocket or by complementary insurance. Again, the insurance system of armed forces and banks covers all the expenses in charity centers.

We initially compared provinces based on the quantity and payment methods of their radiotherapy facilities. The second comparison was based on the ratio of radiotherapy machines to the population of each province, which was calculated as the number of machines per one million people.

In the first step, we mapped the cities with radiotherapy facilities to describe patient proximity to radiotherapy centers in the country. Our next step was to create circles with a 50-mile (80-kilometer) radius around that city's center using Python 3.6.1. A cut-off of 50 miles was selected based on prior publications [11]. Moreover, the data related to radiotherapy staff and facilities in 2022 was compared with 2010 and 2015 in Iran, which were published earlier [8, 12].

#### Evaluation of RT infrastructure and staffing requirements based on guidelines

The incidence rates of all cancers, excluding non-melanoma cancers, in Iran in 2020 were derived from GLOBOCAN [1]. In the next step, The Lancet Commission and Australian publications method were used to calculate the optimal radiotherapy utilization rate (RUR), mainly based on the epidemiological cancer incidence rates and indications of RT for each tumor site [6, 13]. Unlike the European Society for Therapeutic Radiology and Oncology (ESTRO), the ESTRO-Quantification of Radiation Therapy Infrastructure and Staffing Needs (QUARTZ) (14, 15), the ESTRO-Health Economics in Radiation Oncology (HERO) [14, 15], and the international atomic energy agency (IAEA) recommendations, the Lancet Commission guidelines omit re-irradiation and palliative radiotherapy. They believe that less than 10-25% of patients receive a second course of radiation with fewer than five fractions, so they do not remarkably influence the overall estimations [6, 16]. Therefore, in the present study, the number of re-irradiation and palliative radiotherapy cases was not included in calculating the total number of patients who needed radiotherapy in 2020.

Finally, we compared the number of radiotherapy staff and facilities in the country in the current situation with the expected number recommended by the world health organization (WHO), ESTRO-QUARTZ, and IAEA guidelines.

#### Statistical analysis

Data analysis was performed with Excel (Microsoft, Redmond, WA) and Python 3.6.1, and maps were created with the Mapchart online tool (Mapchart.net).

## Results

### RT centers

**Fig. 1:**
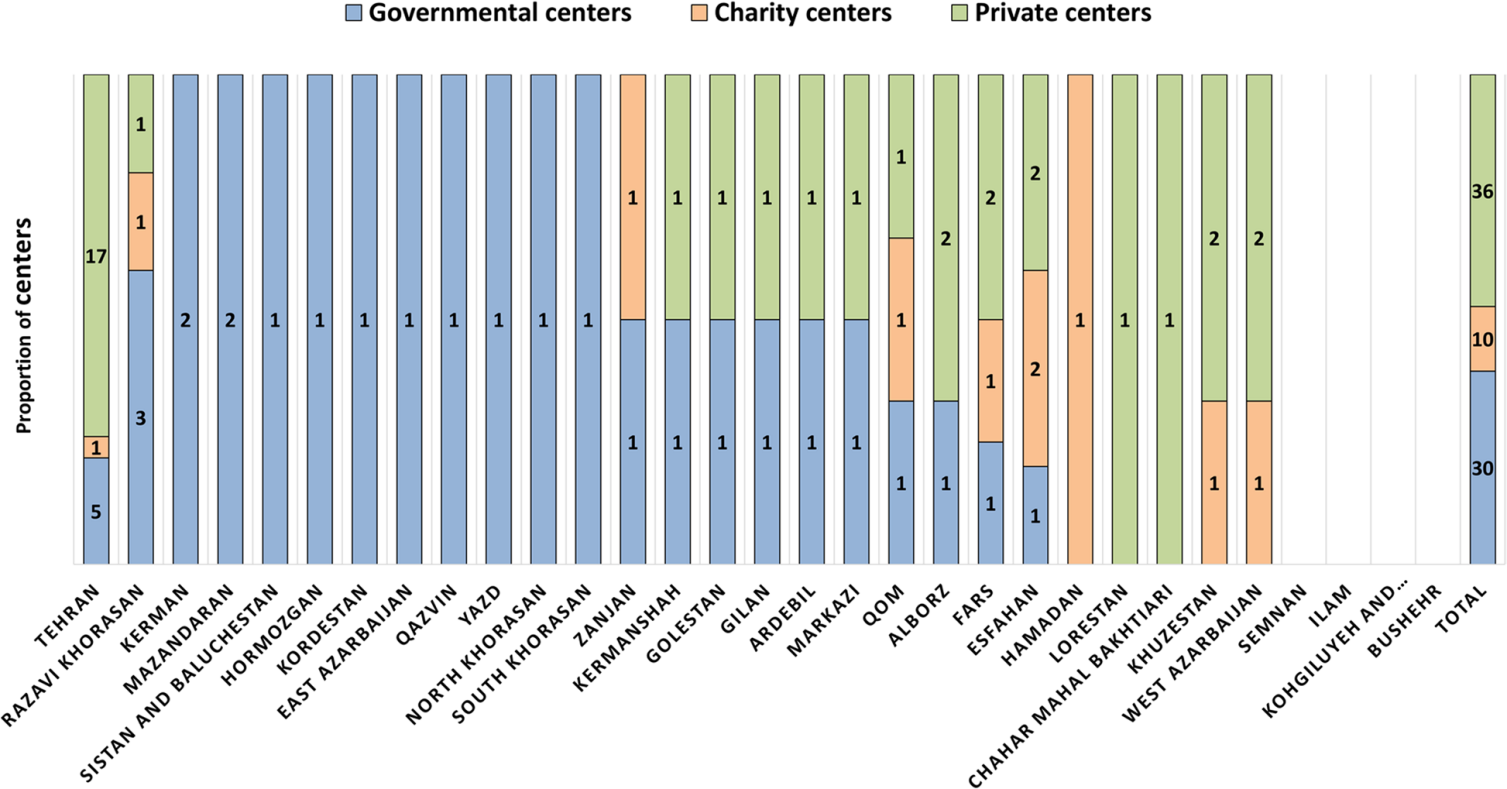
The distribution of public, charity, and private radiation therapy (RT) centers in all provinces of Iran in 2022

Overall, the response rate from RT centers was 100%. A total of 84 RT centers were identified. Seventy-six external beam RT centers were active and treating patients, while eight, all public, were under construction. Overall, out of active facilities, 30 centers were public (39.5%), 10 (13%) were charity-based, and 36 (47.5%) were private. Fig. 1 presents the distribution of each type of RT facility in all provinces.

### Radiotherapy equipment

A total of 123 linear accelerators (LINAC) were identified in this study. The ratio of LINACs to centers was 1.6 (range 1-4), which means that thirty-eight centers (50%) had only one LINAC. The Iran Cancer Institute was the center that possessed the highest number of LINAC machines, which was four, while all the other centers had fewer machines. Computed tomography (CT) simulation was conducted in 30 centers, while outsourced diagnostic CT scans were used in 46 other centers. There were only 4 active, dedicated large-bore CT simulators (two public and two private centers), while all other CT simulations were performed using installed diagnostic CT scan machines. Of diagnostic CT machines, 15 (50%), 13 (43%), and 2 (7%) CT machines belonged to public, private, and charity-based centers, respectively.

Overall, 39 centers (51% of all centers) with 58 LINACs (47% of all LINACs), including 14 public centers (47% of public centers) with 16 LINACs, five charity-based centers (50% of charity-based centers) with seven LINACs, and 20 private centers (56% of private centers) with 35 LINACs had the required capacity and facilities to perform intensity-modulated radiation therapy (IMRT). Only three centers, which were private and LINAC-based, performed stereotactic body radiation therapy (SBRT). From 2002 until 2017, the first Gamma Knife® machine annually treated 1000 patients; however, due to the expiration of its cobalt head, it has remained inactive since 2017. (Table 1). Another brand-new Gamma Knife machine (ICON, Elekta) has been installed in a university hospital in Tehran. It started to treat patients in 2023, which is out of our study period.

**Table 1:** The trend of radiotherapy facilities and human resources based on population of Iran from 2010 to 2022

Variables	**2010**	**2015**	**2022**	**Percentage of changes compared to 2015**
Population (*10^6^)^a^	73.7	78.4	85	+ 8.4%
GNI per capita ($USD)^b^	6250	5710	3370	- 40.9%
Radiotherapy staff				
Radiation oncologist	147	243	344	+ 41.6%
Radiation oncologist / 1 million population	1.99	3.10	4.04	+ 30.3%
Medical physicist	N/A	188	252	+ 34.0%
Medical physicist / 1 million population	N/A	2.39	2.96	+ 23.8%
Radiotherapy technologist	N/A	384	419	+ 9.11%
Radiotherapy technologist/ 1 million population	N/A	4.89	4.92	+ 0.61%
Radiation Oncology Residents	N/A	N/A	165	N/A
Radiotherapy facilities				
Radiotherapy center	34	61	76	+ 24.6%
Radiotherapy machine	32	77	123	+ 59.7%
Radiotherapy machine / 1 million population	0.43	0.98	1.44	+ 46.9%
Brachytherapy machine	N/A	N/A	14	N/A
Number of centers with each radiotherapy machine type				
IMRT/ IGRT	None	None	58	N/A
MLC	N/A	N/A	64	N/A
EPID	None	None	58	N/A
CT simulation	N/A	N/A	30	N/A
IORT	N/A	N/A	4	N/A
Orthovoltage device	N/A	N/A	1	N/A

*GNI* per capita Gross National Income divided by mid-year population, *IMRT* Intensity-Modulated Radiation therapy, *IGRT* Image-Guided Radiation Therapy, *MLC* Multi-Leaf Collimator, *EPID* Electronic Portal Imaging Device, *CT* stimulator: Computed Tomography stimulator, *IORT* Intraoperative radiation therapy (IORT), *N/A* not applicable regarding missing data

^a^Based on the official statistics center of Iran

^b^Based on World bank data

Regarding intraoperative radiation therapy (IORT), four centers including one private and three public possess a machine but none are currently active. The IORT devices were used for breast cancer primarily. There is only one active orthovoltage unit located in a public academic center that treats superficial skin cancers. There are 14 active after-loading machine for high-dose-rate brachytherapy (BT) in 14 centers. Of these, two are private-based and two are charity-based centers that have EBRT machines as well. The remaining 10 machines belong to public academic centers. There is no active low- or medium-dose rate BT machine in Iran.

Up to 2023, no magnetic resonance linear accelerator (MR-LINAC) or proton therapy center in Iran has been installed. Regarding the lifetime of machines, there were no active LINACs with less than five years of production, even those with IMRT/IGRT capabilities. The majority of LINACs had been installed for 10 to 15 years. The mean and median machine working hours per day were 12.73 and 12 (range 7.5 to 20), respectively.

### Radiotherapy staff

There were 344, 252, and 419 active radiation oncologists, medical physicists, and radiation therapy technologists (RTT) in 2022, respectively. Radiation oncologists (now called Radiooncologists), like clinical oncologists in the United Kingdom, Scandinavian, and some Western European countries, are certified to carry out radiation therapy and systemic therapies. As shown in Table 1, there were remarkable changes in the number of radiation oncologists, medical physicists, and RTTs from 2010 to 2022. These changes were consistent with the changes in RT centers and LINACs. The highest rate of increase in radiotherapy staff compared to 2015 was related to radiation oncologists, with 41.6%, and the lowest was related to RTTs, with 9.1%. RTTs in private centers are allowed to work in public centers, too. And according to the findings, these centers employed no dosimetrists. In Iran medical physicists and RTTs have a role in planning and verification. There are also no registered dedicated radiation oncology nurses in Iran.

### Distribution of resources across Iran

**Fig. 2:**
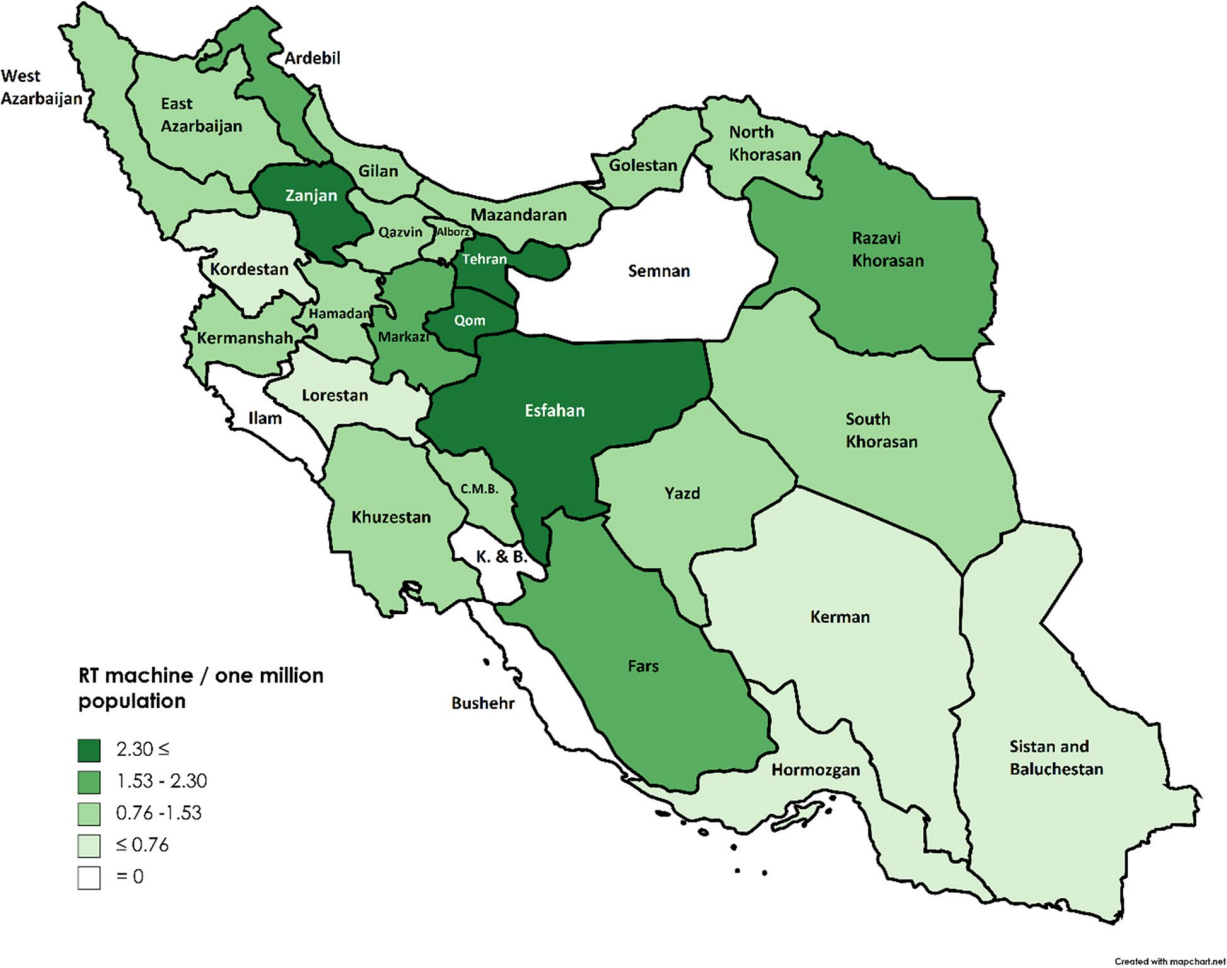
The number of existing radiation therapy (RT) machines per one million population in all provinces of Iran in 2022. K&B: Kohgiluyeh & Buyerahmad. CMB: Chahar Mahal Bakhtiari

The number of LINACs per one million people in all provinces of Iran is presented in Fig. 2. On average, there were 1.53 LINACs per one million people in the entire country. Using this information, the provinces were categorized into four quartiles. Seven provinces were higher than the national average, and 20 were lower. Four provinces, including Ilam, Semnan, Kohgiluyeh-Boyer-Ahmad (K & B), and Bushehr, had no RT facilities. The capital of Iran, Tehran, with a population of nine million, has the highest number of RT centers, 23 centers with 38 LINACs (Fig. 2).

**Fig. 3:**
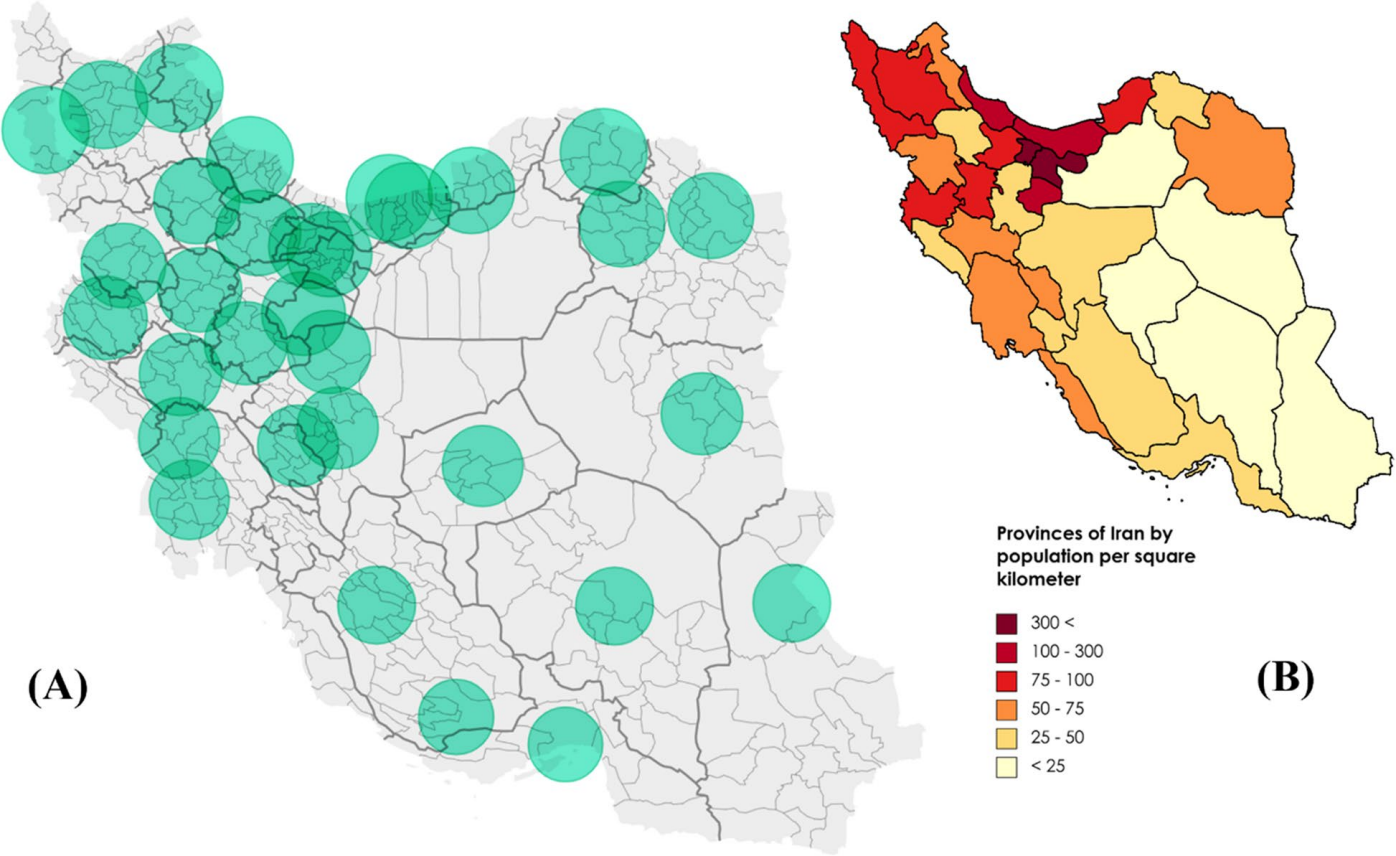
**A** The location of cities with radiotherapy centers with a 50-mile (80 km) radius around them in 2022. **B** Population density of all provinces of Iran in 2022

Figure 3A presents the 80-km Euclidean distance around the cities with radiotherapy facilities. In all provinces, facilities were provided in the capital city, except for five provinces, including Razavi-Khorasan, Mazandaran, Khuzestan, Isfahan, and Fars. The density of centers was higher in the western and northern parts of the country, resulting in more coverage for accessing radiotherapy centers. Also, in closer regions to the capital, this density increases to the extent that their geographical coverage overlaps. As shown in Fig. 3B, the population density is higher in the northern and western parts of the country. The distribution of centers in the country follows the population density. However, as seen in Fig. 3B, patients in the southern and eastern cities of the country need to commute more than 50 miles (80 km) to access radiotherapy services.

### Patient load

As per our survey, there were nearly 58,000 external beam treatment courses documented between September 17, 2021, and September 17, 2022. Based on epidemiological data on cancer prevalence in the latest release of GLOBOCAN and RT indications for each tumor site, the optimal RUR was 0.45 that equals to almost 57,000 patients in 2020 (Table 2). Although reirradiation and palliative radiotherapy have not been included in our computations, according to the Lancet Commission [6], less than 10-25% of patients receive a second course with less than five fractions. Thus, the overall estimations are not remarkably influenced by these treatment courses. Moreover, the likelihood of receiving radiation therapy twice in a patient within a year is particularly low.

**Table 2:** The current and expected number of radiotherapy machines and staff based on various criteria

Resources	**WHO**	**ESTRO-QUARTS**	**IAEA**
Radiation oncologist (criteria)	4 per 1 million population	1 per 250 patients per year	1 per 250-300 patients per year
Current situation (2022)	344	344	344
Expected	340	228	190 - 228
Current / expected	>100%	>100%	> 100%
Medical physicist (criteria)	4 per 1 million population	1 per 450-500 patients per year	1 per 300-400 per patients per year
Current situation	252	252	252
Expected	340	114 - 126	142 - 190
Current / expected	74%	> 100%	> 100%
Radiotherapy technologist (criteria)	8 per 1 million population	---	1 per 100-150 patients per year
Current situation (2022)	419	---	419
Expected	680	---	380 - 570
Current / expected	61%	---	73 – 100%
Radiotherapy machine (criteria)	4 per 1 million population	1 per 450 patients per year	1 per 200-500 patients per year
Current situation (2022)	123	123	123
Expected	340	126	114 - 285
Current / expected	36%	97%	43 – 100%
Population (*10^6^) in 2022 ^a^	85	85	85
Radiotherapy cases estimated in 2020 ^b^	57000	57000	57000
Radiotherapy cases received RT in 2022^c^	58000	58000	58000

*WHO* World Health Organization, *ESTRO* European Society for Radiotherapy and Oncology, *QUARTS* Quantification of Radiation Therapy Infrastructure and Staffing needs, *IAEA* International Atomic Energy Agency

^a^Based on the official statistics center of Iran

^b^based on WHO Global Cancer Observatory Data 2020 (https://gco.iarc.fr/today) and Estimation of number of cases requiring radiotherapy (based on Radiotherapy Utilization Rate 2015 by Atun et al.) excluding palliative and re-irradiation

^c^actual RT courses including palliative and re-irradiation

### Expected number of radiotherapy staff and equipment

Table 2 compares the current situation of radiotherapy staff and equipment according to WHO (12, 19), ESTRO-QUARTZ, and IAEA guidelines [12, 17–19]. Based on all three guidelines, the total number of radiation oncologists is acceptable, but radiotherapy equipment needs to be added. Based on WHO policies, there is a shortage of staff and equipment. However, according to statements from the IAEA, all the available resources were adequate.

### Education

Radiation oncologists in Iran are involved in prescribing both chemotherapy and radiation therapy. With an upward trend, 35±5 residents have been trained each year since the introduction of the new program in 2017. The radiation oncology residency program is competitive and takes five years, with rotating through the internal medicine ward during the first year. Currently 11 universities offer radiation oncology residency program. Fellowship programs including either technique-based or organ-based are yet to be offered. However, some radiation oncologists have been trained in fellowship programs with variable durations abroad.

## Discussion

This study aimed to provide an overview of Iran's current status of radiotherapy human and equipment resources and its challenges in achieving health equity all over the country. In addition, we tried to evaluate the trend of service in comparison to data from 2010 and 2015.

Our results revealed that Tehran, as the capital of Iran, with 10% of the national population, had the highest number of RT governmental centers, which accounts for 16% of all the country's governmental centers. In contrast, five provinces, with 15% of the country's total population, had no governmental centers. According to the 2016 census, 10.3% of Iran's population is entirely uninsured [20], and only 10% have supplementary health insurance [21], while one-third of RT resources are private. This issue could increase patient congestion in public centers, increasing the average wait time for RT services. Also, the load of patients has led to the non-adherence of physicians to hyper-fractionated regimens when indicated. Moreover, waiting lists for definitive therapy and palliative care can compromise clinical outcomes and quality of life [8]. It seems that the lack of universal health coverage and policies of reimbursement systems in the country are among the contributing factors to inequity in healthcare delivery.

Our findings implied that the unequal geographic distribution of RT centers (and LINACs) across the country might lead to disparities in providing care. Provinces with higher economic status, which could cover the cost of the private RT centers, had better availability of RT services, as most had more than 2.3 RT machines per one million province population. On the other hand, areas with economically weaker conditions had the poorest delivery of RT services, as some had below 0.7 RT machines per one million population, and even four had no RT facilities at all. In some centers, mainly located in the Eastern and Southern regions, there may be difficulties when the only LINAC machine is out of order.

Previous research emphasized that difficult access and long travel distances cause a financial burden, forcing some patients to withdraw from treatment or visit physicians in later stages, yielding poorer outcomes [22, 23]. Based on the present study, it was supposed that Iranian residents, particularly those residing in the eastern, southern, and southeastern regions, have a lower likelihood of receiving RT services as they live more than 50 miles (80 km) away from the centers.

According to the findings, 84 RT centers, consisting of 123 LINACs, were identified in the country. Besides, 344, 252, and 419 active radiation oncologists, medical physicists, and radiation therapy technologists (RTT) were identified in 2022, respectively. Establishing appropriate guidelines for radiation therapy can be challenging due to variations in cancer incidence and trends across different countries, as well as differences in workload, such as the amount of time machines and staff are available per shift. Additionally, treatment duration may differ between centers within a country and between low- and high-resource countries [15]. Besides, since diverse staff roles and responsibilities exist between countries, defining the required number of radiation specialists is complex [15]. Most high-income countries have provided precise data on RT staffing, and there needs to be more data in low- and middle-income countries [6, 15]. The guidelines for the required number of RT human and equipment resources are mainly based on the situation in European countries [18]. That is why QUARTZ has issued a range with no definitive recommendations [18]. Although Iran's current status regarding human and equipment resources is relatively acceptable based on the QUARTZ recommendations, the countries in the neighborhood have limited infrastructures per one million as it is 0.28 in Pakistan and indeterminate for Afghanistan [24] and Iraq [25]. Iran is hosted for some patients in border areas whose definite number is unknown. It should be borne in mind that radiation oncologists in Iran are involved in prescribing both chemotherapy and radiation therapy.

Iran has recently fallen from a high-middle-income country to a low-middle-income country (LMIC), owing to a 40.9% decline in the gross national index (GNI) per capita from 2015 to 2022 [26]. Despite the remarkable drop in GNI per capita and the increase in population, a rising trend can be seen from 2010 to 2022 regarding RT facilities and staff. Although, after omitting the palliative and re-irradiation that roughly comprise 10% of treatment courses in our domestic reports [27], about 91.5% of patients who needed RT has received it in our survey. However, these estimates only reflect the quantity of treatment courses and not the quality of the techniques of planning and delivery of RT to the patients. Thus, considering the age and capability of available equipment, there are concerns regarding the need for more quality RT services in the near future, especially advanced techniques including IMRT/VMAT and SBRT. In Iran, modern RT treatments are limited due to a shortage of trained radiation oncologists, personnel, and equipment maintenance. Aside from developing RT machines and techniques, proper planning is necessary to increase the expertise of professionals in complex and advanced RT delivery and planning techniques.

Iran’s need for RT based on optimal RUR is close to other countries like Netherlands, Columbia and Argentina, Nigeria and Egypt [1]. Considering the number of LINACs in these countries our situation is more like Egypt that is better than Columbia and Nigeria but worse than Argentina and Netherlands [28]. It should be noted that these estimates only reflect the number of RT machines and not the quality of services and techniques.

Estimation for BT services, orthovoltage, and IORT has not been addressed in specific guidelines and was not available in ESTRO-QUARTZ, IAEA, or the Lancet Commission recommendations. Brachytherapy is one of the necessary modalities in delivering radiation to patients, particularly for cervix and prostate, head and neck, and skin cancer. Application of BT accounts for up to 10% of total radiotherapy treatment courses in the USA and 25% in European countries [29]. It’s worth noting that, despite high demands for BT services due to the high incidence of cervical cancer in Iran [4], the lack of specialized staff and dedicated wards has caused the infrastructure to be insufficient in this regard.

This study has some limitations. Firstly, in the current study, to calculate the optimal RUR based on the Lancet Commission and Australian publications, the epidemiological cancer incidence rates and indications for each tumor site method were used [6, 13]. However, variable patterns of cancer presentation make it challenging to estimate the proportion of cancer cases requiring RT. The RUR can be derived from epidemiological/evidence-based or criterion-based guidelines [6, 30]. Epidemiological/evidence-based guidelines have estimated higher rates of maximum demand as they consider the type and stage of cancer. Also, in developing countries, empirical approaches are difficult to define due to a lack of comprehensive patient data resources and long-term follow-up [31]. Secondly, since the accuracy of cancer incidence rates obtained from the GLOBOCAN varies depending on the country's health system, registry, and income status, the accuracy of the calculations might have been affected. However, according to GLOBOCAN, Iran has been classified as one of the 19 countries with high-quality incidence data. Third, it is noteworthy that the GLOBOCAN estimates do not reflect the effect of the coronavirus disease 2019 (COVID-19) pandemic [1, 32]. The complete scope of the impact of COVID-19 is still unknown. However, delays in diagnosis, treatment, and follow-up because of public concerns, screening program closures, and limited access to healthcare systems are expected to increase cancer incidence [32–34]. In addition, the available formulations and estimations of RUR do not identify the technique of RT delivery or guidance. As we know, many head and neck or central nervous system cancers or malignancies in the pediatric population require advanced and sophisticated techniques, including SBRT/SRS, IMRT/VMAT, MR-LINAC, and Proton therapy, that are either unavailable in Iran or severely scarce throughout the country. Another limitation would be our lack of information regarding altered fractionation schedules. We know that one appropriate way to shorten the waiting list is hypofractionation when evidence supports such a fashion.

## Conclusion

Although there has been notable growth in RT facilities in recent years, and Iran's current situation is acceptable as an LMIC country, it still needs more work to reach an acceptable level. To improve the country's RT status, qualitative goals such as the appropriate distribution of active facilities considering the regional cancer incidence rate, increasing the expertise of related professionals, and quantitative goals of providing more modern radiation delivery in the future, seem necessary.

## Data Availability

Data would be available based upon an eligible request to the corresponding author.

## Abbreviations

RT: Radiation therapy
RORC: Radiation Oncology Research Center
IORT: Intraoperative radiation therapy
RUR: Radiotherapy utilization rate
ESTRO: European Society for Radiotherapy and Oncology
ESTRO: European Society for Therapeutic Radiology and Oncology
QUARTZ: Quantification of Radiation Therapy Infrastructure and Staffing Needs
HERO: Health Economics in Radiation Oncology
IAEA: International Atomic Energy Agency
WHO: World Health Organization
CT: Computed tomography
LINAC: linear accelerators
IMRT: Intensity-Modulated Radiation Therapy
SBRT: stereotactic body radiation therapy
RTT: Radiation therapy technologists
LMIC: Low-middle-income country
GNI: Gross National Income
